# Distinction between Nausea and Vomiting

**Published:** 1813-09

**Authors:** 


### *To the Editors of the Medical and Physical Journal.*

GENTLEMEN,

THE jealousies and dissensions which exist between medical men cannot be too highly censured, since they are truly disgraceful to a profession which is confessedly of the most liberal kind. A man of eminence who dares to publish to the world an important discovery, and which bids fair more effectually than any other to restrain the ravages of disease, will perhaps risk the loss of that reputation which his superior acquirements have justly attained for him, by the interference of an officious contemporary, who, in the plenitude of his zeal, vociferates that either he has previously made use of the same remedy for the same disease, or that it may be met with in some ancient or modern publication.

I was particularly led to these observations by reading a paper, in your excellent Journal for June, on the utility of Nausea in Ophthalmia and other Diseases of the Eye, by Mr. Fielding of Hull. This gentleman sets out with an encomium on Mr. Adams's method (which is inserted in a former number), but apparently only with the view of introducing his own, and thus, as he seems to think they are in every respect similar, he takes away from Mr. Adams the merit of originality.

It is foreign from my present purpose to make any remarks on the rationality of the practice recommended by either of these gentlemen, but it appears evident to me, that if the same effect be brought about by nausea as by active vomiting, it must be the product of causes diametrically opposite to each other. In order to substantiate this assertion, it is necessary for me to consider the effects of nausea and of vomiting on the constitution, and thus, by contrasting them, point out their dissimilarity.

Nausea, by acting primarily on the stomach, seems, as a consequence of that action, to depress very materially the powers of the whole system. The patient, even if he be of the most athletic make, and consequently previous to the administration of the nauseating matter could have made effectual resistance to the most powerful efforts of restraint, becomes deprived of all muscular energy, occasioning the highest degree of prostration of strength and listlessness, so that he is not only unable but also unwilling to struggle for liberty, if even such were necessary. The same general cause represses, to a certain degree, the action of the heart and arteries, which, being thus rendered incapable of propelling pelling their contents onward to the extremities, give to the surface of the body (especially the cheeks and lips) a pallid death-like appearance; and I make no doubt that if the patient be at this time laboring under ophthalmia, and the vessels of the conjunctiva injected with red blood, they also will be emptied of their contents, and will put on their naturally healthy appearance. In short, the effects of nausea are a relaxation of the voluntary muscles, and also of the involuntary so far as the heart and arteries are concerned.

Could the degree of nausea be carried to an indefinite extent, I make no doubt but the powers of life would be ultimately extinguished by it; but here nature seems to have placed an effectual barrier, for by increasing the nauseating matter, instead of increasing the nausea we excite vomiting, and thus effects very opposite to those I have been describing are produced; for now, instead of a relaxation of the muscles, instead of a languid circulation and a death-like listlessness, nature is roused to make a powerful effort to get rid of the offending matter; the muscles, not only of respiration, but also of the extremities, are thrown into strong contractions, and the heart and arteries propel their contents onward to the ultimate branches with increased velocity; so that it frequently happens, during violent vomiting, the delicate vessels of the conjunctiva covering the sclerotic coat are ruptured, and blood consequently extravasated into the cells of the reticular membrane, constituting what is called a blood-shot eye; the pulse, instead of being so quick and feeble as scarcely to be felt or counted, as we find it to be during nausea, now bounds under the finger; and the face, partly owing to the pressure of the contracted muscles on the veins hindering the return of the blood through them, and partly to the increase of circulation, is suffused with a dark crimson hue, and apparently swoln, instead of being flaccid and deathly pale.

Having thus endeavored to contrast the different effects of nausea and of vomiting, I shall briefly conclude by asking, where is that similarity of action between them which can justly give rise to the following conclusion—because one of them is a useful remedy for a particular disease, the other, acting on the same principle, must also necessarily be so? The only difference is, that the latter is more decisive in its effects, because it is more powerful in its operations.

I remain, Gentlemen,

Your obedient Servant,

C. W. S.

*Clifton, Bristol,*

